# Zoonotic and reverse zoonotic transmission of severe acute respiratory syndrome coronavirus 2: a review and implications for Africa

**DOI:** 10.11604/pamj.2021.38.39.24170

**Published:** 2021-01-14

**Authors:** AbdulAzeez Adeyemi Anjorin, Oluwaseyi Sedowhe Ashaka, Sodiq Olawale Tijani, John Oluwayomi Openibo, Ismail Ayoade Odetokun

**Affiliations:** 1 Department of Microbiology (Virology Research), Lagos State University, Ojo, Lagos, Nigeria,; 2 Department of Medical Microbiology and Parasitology, College of Health Sciences, University of Ilorin, Ilorin, Nigeria,; 3 Department of Medical Microbiology and Parasitology, College of Medicine, University of Lagos, Idi-Araba, Nigeria,; 4 Department of Biological Sciences, College of Science and Technology, Covenant University, Ota, Ogun State, Nigeria,; 5 Department of Veterinary Public Health and Preventive Medicine, University of Ilorin, Ilorin, Kwara State, Nigeria

**Keywords:** Coronavirus, SARS-CoV-2, COVID-19, zoonotic and reverse zoonotic transmission, Africa

## Abstract

There is an urgent need to properly understand the transmission dynamics of severe acute respiratory syndrome coronavirus 2 (SARS-CoV-2) in the event of continuous rise in morbidity in both humans and animals as well as an increase in the mortality rate in man. Since the novel SARS-CoV-2 emerged in Wuhan, China with its global spread in over 200 countries, several studies have been published on the epidemiology of the virus in man with limited information on the roles of animals and the possibility of reverse zoonosis. We therefore collected published research literature on COVID-19 from public search engines for information on SARS-CoV-2 in animals and reverse zoonosis from man. A critical and thorough study appraisal/evaluation was performed to include recent quality publications that focus on the scope of this write-up including zoonosis and reverse zoonosis of SARS-CoV-2. We highlighted what is known about SARS-CoV-2 in animals, identify gaps for future research, summarized possible reverse zoonotic transmission of SARS-CoV-2 from man to animals and included the likely implications of our summary for Africa, despite the dearth of information in Africa on the key concepts of this study.

## Introduction

Coronavirus (CoV) was first isolated in birds in 1937, 6 years after it was initially described in 1931. The first human CoV (HCoV) was isolated in human embryonic tracheal organ culture in 1965 [1-3]. After more than five decades, a novel coronavirus with a pandemic potential was isolated from humans at the end of 2019 which was named severe acute respiratory syndrome coronavirus 2 (SARS-CoV-2) by the International Committee on Viral Taxonomy Coronaviridae Study Group (ICTV-CSG) on the 11^th^ February 2020, the same day the disease caused by this virus was named Coronavirus Disease 2019 (COVID-19) by WHO [4,5].

Two classical groups of HCoVs exist: the high/severe HCoVs (SARS-CoV-1), which include the Middle East Respiratory Syndrome Coronavirus (MERS-CoV) and SARS-CoV-2, that belong to the alpha and beta CoVs causing lethal respiratory infections; and the low/seasonal HCoVs (HKU1, HCoV-OC43, HCoV-NL63 and HCoV-229E) classified in the gamma and delta CoVs, causing self-limiting infection [6,7].

Several animals harbour viruses with ecological importance due to the dynamics of their transmission to humans. For instance, rodents transmit Lassa fever virus, water fowl serves as the reservoir for influenza, while bats harbour nipah virus and SARS-CoV-2. Man, at the receiving end occasionally returns the gesture back to animals. Therefore, zoonosis and reverse zoonotic transmission (known as zooanthroponosis) remain two major concepts at the human-animal interface. The understanding of the origin and transmission dynamics among humans and animal species will aid the control efforts and guide further interventions in the control of COVID-19 especially in Africa where there is limited biosecurity control. Bats have been reported as the natural reservoir for SARS-CoV-2 which has caused infections in humans [8]. Pangolins, dromedary camels and civet cats have been identified as intermediate host for other coronaviruses that has plagued man [9-13]. It has also been opined that genetic data refutes the claim that SARS-CoV-2 emerged from laboratory manipulations. This has been explained through two scenarios which are genetic fitness of the virus in an animal host prior to zoonotic transmission and genetic fitness of the virus in humans following zoonotic transmission [14].

The source of the initial cases of COVID-19 pandemic linked with China wet seafood market in Wuhan, China suggests a possible zoonotic transfer from animal species to humans [15,16]. This is because a multitude of small and large domestic and wild animals are offered for sale and human consumption at this market [15,17-24]. Lately, SARS-CoV-2 was detected in several animal species such as pangolins, cats and ferrets and some zoo animals.

There is still a lot of information gap when SARS-CoV-2 is being considered and these include the possibility of human to animal transmission. Most of the published reports are outside the shores of Africa. The scarcity of these reports in Africa does not obscure the possibility of the transmission of this virus from humans to animals elsewhere. Recently in Hong Kong, two dogs, a Pomeranian and a German shepherd were infected with SARS-CoV-2 from infected persons [9]. The wildlife conservation society also confirmed the infection of tigers with SARS-CoV-2 in the Bronx zoo in New York City after being exposed to an infected zoo keeper [25]. In line with this potential risk, the WHO has advised the public to avoid unprotected It is no longer news that significant proportions of human population worldwide including Africa are infected and being infected with SARS-CoV-2. Infected individuals are at the risk of spreading the virus unconsciously before the manifestation of symptoms or in asymptomatic condition thereby increasing the risk of transmission of SARS-CoV-2 to domestic animals and livestock in Africa. Some reports exist about SARS-CoV-2 transmission from human to contact with both farm and wild animals because of their roles in the pandemic [5].

In Africa, it is pertinent to avoid establishment of animal reservoirs which could increase the risk of recurrent re-infection in humans and jeopardize the SARS-CoV-2 control efforts being struggled within the continent. Although, the current body of evidence that are available are too few to make substantive conclusions in Africa. However, this review looked into the natural and experimental infections in animals as well as the prediction of viral interactions taking into perspective the peculiarity of Africa. It specifically highlighted some of the implicated animals and the role of man in the reverse transmission of SARS-CoV-2 to these domestic and non-domestic animals with a few highlighted suggestions to prevent the occurrence of such a phenomenon on the Africa continent (Table 1).

**Table 1:** a summary of key findings on zoonotic and reverse zoonotic transmission of SARS-CoV-2

S/N	Key findings
1	Isolation of the first coronavirus in bird in 1937
2	SARS-CoV-2 was named by the International Committee on Viral Taxonomy (ICTV) on the 11th February, 2020
3	The disease caused by SARS-CoV-2 was named coronavirus disease 2019 (COVID-19) by WHO on the 11th February, 2020
4	Bats are natural reservoir for SARS-CoV-2
5	Pangolins, dromedary camels and civet cats were identified as intermediate host for other coronaviruses
6	A Pomeranian and a German shepherd dogs were infected with SARS-CoV-2 from infected persons (reverse zoonosis)
7	Tigers infected with SARS-CoV-2 by zoo keepers in Bronx zoo in the New York City, USA
8	Genus rhinolophus of bats commonly found in Africa, Asia and Europe habour SARS-CoV-2
9	SARS-CoV-2 found in infected domestic cats and dog in Hong Kong and Belgium
10	Evidence of SARS-CoV-2 transmission from cats to cats
11	Ferrets were observed to be susceptible to SARS-CoV-2 and could spread the virus directly or indirectly
12	Malayan tiger showed signs of respiratory illness for positive SARS-CoV-2 with human-animal spread from a zookeeper in Bronx Zoo, New York, USA. Lions were infected with coronavirus in Bronx Zoo
13	Mink from multiple farms were positive for COVID-19 with spread among minks. Zoonosis was reported from mink to humans
14	Reports of coronavirus infections in animals like birds, swine, cattle, camels, horses, dogs, cats, bats, rodents, minks, palm civets, rabbits, ferrets, snake and several other wild animal sexist

These are reports as at August, 2020

## Methods

**Figure 1:**
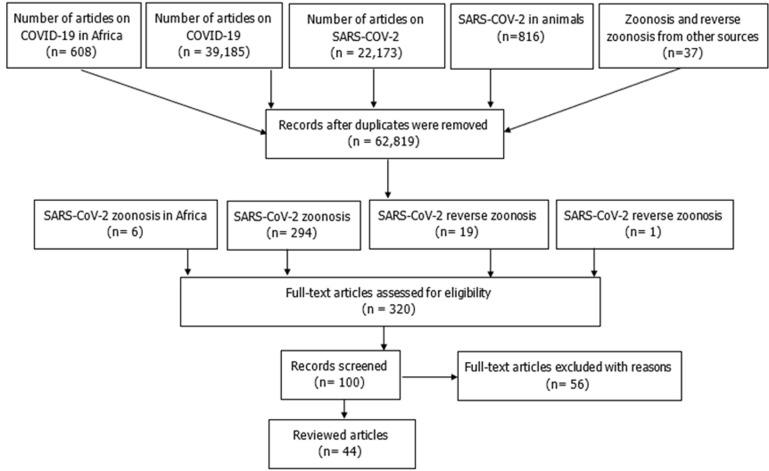
flow chart of selected articles for review

We explored published literatures on COVID-19 from the global public repositories including Google, Google scholar and Pubmed for information on SARS-CoV-2 in animals and documented evidences of reverse zoonotic transmission from man using search terms and words like “SARS-CoV-2”, “reverse zoonosis of SARS-CoV-2”, “SARS-CoV-2 in animals”, “cases of coronavirus in animals”. Also, information were obtained from the databases of the World Health Organisation (WHO), World Organisation for Animal Health (OIE), Wildlife Conservation Society, the United States Department of Agriculture and the American Veterinary Medical Association. The literatures were critically scrutinized and evaluated to include recent and quality publications that focused on the scope of this write-up as part of our inclusion criteria to majorly review studies on zoonosis and reverse zoonosis of SARS-CoV-2. Relevant articles with useful suggestions for Africa were also included (Figure 1). Abstracts, conference proceedings and unpulished studies or articles with low relevance to the scope or target of this study were excluded. This was followed by a thorough study appraisal for information syntheses. However, this study added the likely implications of zoonosis and reverse zoonosis of SARS-CoV-2 on Africa despite the limited/dearth of information on the topic in the continent.

## Current status of knowledge

**Zoonotic transmission of SARS-CoV-2 in animals:** laboratory and genomic analyses have revealed a 96.2% similarity between bat coronavirus and SARS-CoV-2 [16,26]. Thus, bats have been incriminated to carry the SARS-CoV-2, but its possibility of direct transmission to human remains obscured hence intermediate hosts are needed for the viral transmission [27]. The SARS-CoV-2 shares similar lineage and certain characteristics with the horseshoe bat and pangolin beta coronaviruses. However, it has been observed that the bat genus rhinolophus carry almost all SARS-like viruses with only a few exceptions. Genetic sequence analyses between SARS-CoV-2 and bat beta coronavirus revealed the circulation of SARS-CoV-2 ancestors in the genus rhinolophus of bats which are commonly found in Africa Asia and Europe [28]. More research is needed to explore the high diversity of the coronaviruses found in bats [15] especially as it relates to Africa with huge bat populations. The SARS-CoV virus has been confirmed in some animals (Table 2). Apart from bats, the Malayan pangolin was also incriminated as a probable intermediate host of SARS-CoV-2 after the coronavirus isolates from the pangolins were found to carry certain genetic characteristics with high similarity to the SARS-CoV-2 [12,29]. However, a study observed a lower similarity between betaCoV/bat/Yunnan/RaTG13/2013 virus and SARS-CoV-2 isolated from infected humans. This makes the pangolin a less likely intermediate host for the zoonotic spread of the SARS-CoV-2 [21].

**Table 2:** implicated animals in the transmission of SARS-CoV-2

Animals	*Total number of cases	Mode of transmission	Clinical signs and symptoms	Country
Dog	5	Reverse zoonosis	Mild illness, breathing difficulties	China, USA, Netherlands, USA
Tiger	5	Reverse zoonosis	Cough and mild respiratory illness	New York city, USA
Cat	20	Reverse zoonosis	Fever and upper respiratory illness; chronic heart condition and hypertrophic cardiomyopathy; gastrointestinal and respiratory disease	China, Spain, Russia, Netherlands, Germany, France, USA, Belgium
Lion	3	Reverse zoonosis	Mild respiratory illness	USA
Pangolin		Reverse zoonosis	No symptoms	Malaysia
Mink	Multiple	Zoonosis and reverse zoonosis	Gastrointestinal and respiratory disease, death	Netherlands
Ferret		Reverse zoonosis	No symptoms	USA
Egyptian fruit bats		Reverse zoonosis	No symptoms	Egypt
Golden Syrian hamsters		Reverse zoonosis	Mild respiratory illness	Syria
Macaques (macacafascicularis and m. mulatta)		Reverse zoonosis	Mild respiratory illness	

* Number as at August 2020

Cases of infection of pets, mainly cats and dogs, have been reported in Hong Kong and Belgium [9,30]. The Pomeranian and the German shephard breeds of dogs were those that got infected. The infection in dogs was regarded as weak as the dogs were able to elicit immune response against the SARS-CoV-2 [15]. Also, under experimental conditions, cats were found to be asymptomatically infected with the SARS-CoV-2 with evidence of transmission to other susceptible cats in China [11,31]. In the same experiment, ferrets were observed to be susceptible to SARS-CoV-2 and could spread the virus directly or indirectly [32]. A big cat, the Malayan tiger housed in the Bronx Zoo, New York City, USA showed signs of mild respiratory illness and tested positive for SARS-CoV-2 with human-animal spread from a zookeeper [33]. Also, some lions were infected with the coronavirus at the same Bronx zoo in New York, USA [34]. Recently in the Netherlands, cases of respiratory illness and mortality were noticed in mink populations. Testing and laboratory analyses showed that the mink from multiple farms were positive for COVID-19 with spread among mink observed. Furthermore, possible spreads from mink to employees have been reported in Netherlands [35].

**Possibility of human to animal transmission of SARS-coronavirus 2:** a serological investigation carried out among cats revealed that there was evidence of serum neutralizing antibodies which indicated that they were infected during the SARS-CoV-2 outbreak in Wuhan, China [13]. There exist reports that addressed the presence of viral RNA and antibodies of SARS-CoV-2 in dogs, tigers, lions and American minks [36]. The reality of SARS-CoV-2 infection in farms and domesticated animals is a concern in African communities where there is increased transmission of SARS-CoV-2 among humans. Therefore, owing to the challenges of mass testing efforts in the human population in Africa, testing of animal species to investigate this outbreak may be far from being a reality. Reports of coronavirus infections in animals like birds, swine, cattle, camels, horses, dogs, cats, bats, rodents, minks, palm civets, rabbits, ferrets, snake and several other wild animals exist in different countries [37,38] with lack of information in Africa. Therefore, the possibility of transmission to these animals in the aftermath of any genetic mutation cannot be entirely ruled out. Although, there are evidences of experimental infection with SARS-CoV-2 in different animals, these cannot totally be relied upon due to limited numbers of such studies and exceptions observed when other closely related coronaviruses that have infected man are considered.

In China, a study by Shi *et al*. [11] conducted direct inoculation of SARS-CoV-2 in some selected animals. This study showed evidence of high susceptibility among ferrets and cats whereas dogs were observed to have a low genetic susceptibility, while chickens, ducks and pigs were not susceptible at all. Further experimentation observed that cats could transmit the virus via respiratory droplets which could pose threat to the control of this virus. During the earlier SARS-CoV-1 outbreak, transmission from humans to animals was also observed from human beings to pigs in China [39]. Although, this level of susceptibility has been refuted by Shi *et al*. [11] in the current SARS-CoV-2 pandemic. Tiwari *et al*. [15] suggested investigation into farms where several animal species are reared to justify the origin of SARS-CoV-2. In the same thought, investigation of farms and animals which showed evidence of human transmission and could be probable reservoir host should be investigated in high burden areas in Africa. However, in this proposed studies, viral adaptation in transmissibility and pathogenesis should be considered.

Andersen *et al*. [14] has discussed proposed theories of genetic fitness of the virus in humans after transmission fromanimalstohumans and subsequent viral adaptation as a result of transmission from humans to humans. In the event of reverse transmission from humans to animals, natural selection in animal host could represent processes in the preparation of a reservoir host that could cause spikes in transmissions after the pandemic has been controlled. The concern is that Africa is blessed with animals that could be involved in the transmission chain of SARS-CoV-2. The presence of coronaviruses in diverse animal host shows that several animal species could harbour these pathogens. The striking ability of SARS-CoV-2 to infect a broad range of distant mammals with only a few animal species having severe infection favour this ideology [14,40].

It is proposed that even if the emergence of a reservoir host is not achieved, there is increased likelihood of genetic recombination occurring among animal species that are susceptible to the SARS-CoV-2 infection thereby constituting a mixing vessel for the evolution of a new virus [41]. In Africa, the transmission of human pathogens to animals may occur more frequently than we are aware of, this is because of the impact of agricultural practices and urbanization which could be the breeding ground for viral epidemics in the future. However, preventive actions must be taken including adequate biosecurity measures at the farm level, interstate collaboration and coordination among African countries, strategic policies to address such, programs to further strengthen the continuous training of public health officials including more virologists and veterinary experts and preventive education for local farmers in the African rural settings, devising cheap methods of producing locally made and readily available personal protective equipment on the African continent, use of multimedia prevention campaign especially by engaging the community leaders [41-43] in order to prevent a repeat of what happened in other parts of the world with reverse zoonotic transmission of SARS-CoV-2 from infected persons to animals [44] and to avoid the possibility of the next viral spill-over from animals to humans.

In addition, we hereby propose: the need to intensify sentinel active surveillance of coronavirus and other aetiologies of deadly diseases following one health approach and possible institution of such practice with more dedicated research fund contributions from every country in the African Union, for researchers in the continent as it is done in the European Union and elsewhere; expansion of testing facilities that will inturn facilitate prompt diagnoses and early report of cases for immediate and adequate intervention before spread; the need for an integrated disease reporting and warning system following the international society for infectious disease promed/epicore model, creation of genomic library and databank repository across the regions for adequate information sharing and disease early-warning signal. Africa must urgently expand her institutional facilities and technological know-how for infection control practices (IPCs), drug production and vaccine development, with a well planned partnership with known international donor agencies for purposeful advancement of her technology against the current and any feature public health threats.

## Conclusion

Human-animal transmission of SARS-CoV-2 remains a huge concern due to the constant interactions between man and animals in the environment. The virus has now been reported in different animal species including pangolins, cats and dogs, tiger and minks, which gives an evidence of the ability of SARS-CoV-2 to successfully infect and adapt to such hosts. Understanding the origin and reservoirs of SARS-CoV-2 are vital to the epidemiology and control of the current COVID-19 pandemic. Therefore, there is need for Africa to become aware of this risk and organize possible public heath tools to prevent reverse spill-over of SARS-CoV-2 from humans to animals and zoonotic transmission back to man.

### What is known about this topic

- It is known that two classical groups of human coronavirus exist: the high human coronavirus causing severe infection and the low human coronavirus causing self-limiting infection;
- Several animals harbour viruses with ecological importance due to the dynamics of their transmission to humans and also, man occasionally return the gesture back to animals. Bats are natural reservoir for viruses causing infections in humans while pangolins, dromedary camels and civet cats serve as intermediate host for other coronaviruses that have plagued man;
- It has been established that coronavirus infections in animals like birds, swine, cattle, camels, horses, dogs, cats, bats, rodents, minks, palm civets, rabbits, ferrets, snakes and several other wild animals exist in other continents with limited or lack of information in Africa.

### What this study adds

- The study pointed to zoonosis and reverse zoonotic transmission as two major concepts at human-animal interface with natural and experimental infections in animals as well as the prediction of viral interactions taking into perspective the peculiarity of Africa;
- It specifically highlighted some of the implicated animals and the roles of man in the reverse zoonotic transmission of SARS-CoV-2 to domestic and non-domestic animals and also suggested the need to investigate farms, zoological gardens and domestic animals in order to prevent the occurrence of such a phenomenon on the African continent;
- The study also showed that there is need for Africa to be fully aware of the risk involved in the transmission of SARS-CoV-2 especially in rural communities which could be the breeding grounds for viral epidemics in the future.
